# Using risk-tracing snowball approach to increase HIV case detection among high-risk populations in Cambodia: an intervention study

**DOI:** 10.1186/s12879-017-2790-1

**Published:** 2017-10-18

**Authors:** Srean Chhim, John Macom, Chettana Pav, Nirada Nim, Phearun Yun, Sopheap Seng, Kolab Chhim, Sovannary Tuot, Siyan Yi

**Affiliations:** 1 FHI 360, Phnom Penh, Cambodia; 2 Asia Pacific Regional Office, FHI 360, Bangkok, Thailand; 3 Chhouk Sar Association, Phnom Penh, Cambodia; 4 KHANA Center for Population Health Research, Phnom Penh, Cambodia; 5 Center for Global Health Research, Touro University California, Vallejo, CA USA; 6 Boeung Keng Kang III, No. 3, Street 330, Chamkarmon, Phnom Penh, Cambodia

**Keywords:** HIV, Risk-tracing snowball approach, Key population, New case detection, Intervention study, Cambodia

## Abstract

**Background:**

Early HIV diagnosis and initiation onto antiretroviral therapy may prevent ongoing spread of HIV. Risk Tracing Snowball Approach (RTSA) has been shown to be effective in detecting new HIV cases in other settings. The main objective of this study is to evaluate the effectiveness of RTSA in increasing the rate of newly identified HIV cases among high-risk populations. Our second objective was to evaluate the effectiveness of RTSA, as compared to the walk-in group, in increasing the number of HIV tests and early case detection.

**Methods:**

This study was conducted from April 1 to September 30, 2016 at two NGO clinics in Phnom Penh, Cambodia. Respondent driven sampling method was adapted to develop RTSA to reach high-risk populations, including key populations and the general population who have social connections with key populations. Bivariate and multivariate logistic regression analyses were conducted.

**Results:**

During the implementation period, 721 clients walked in for HIV testing (walk-in group), and all were invited to be seeds. Of the invited clients, 36.6% agreed to serve as seeds. Throughout the implementation, 6195 coupons were distributed to seeds or recruiters, and resulted in 1572 clients visiting the two clinics with coupons (RTSA group), for a coupon return rate of 25.3%. The rate of newly identified HIV cases among the RTSA group was significantly lower compared to that in walk-in group. However, the highest number of newly identified HIV cases was found during the implementation period, compared to both pre- and post-implementation period. Although statistically not significant, the mean CD4 count of newly identified HIV cases detected through RTSA was almost 200 cells/mm3 higher than that in the walk-in group.

**Conclusions:**

Although the rate of newly identified HIV cases among the RTSA group was lower than that in the walk-in group, the inclusion of RTSA in addition to the traditional walk-in method boosted new HIV case detection in the two participating clinics. A higher mean CD4 count for the RTSA group may reveal that RTSA may be able to detect HIV cases earlier than the traditional walk-in approach. Further research is needed to understand whether RTSA is a cost-effective intervention to prevent ongoing spread of the HIV among high-risk populations in Cambodia.

## Background

Over the past two decades, Cambodia has been successful in its fight to slow the spread of HIV [1]. HIV prevalence among the general population decreased from an estimated 1.6% in 1998 to 0.6% in 2015 [2, 3]. The estimated number of new HIV cases per year also decreased from roughly 4400 in 2005 to 700 in 2015 [2, 3]. Despite this success, as of 2015, an estimated 74,000 Cambodians were living with the virus [4]; with about 27% of these individuals (~19,000) being unaware of their HIV-positive status [2].

Early HIV diagnosis and initiation onto antiretroviral therapy (ART) may prevent ongoing spread of HIV. Knowledge of one’s HIV-positive status has been shown to be associated with increased condom use [5]. ART initiation, when resulting in viral load suppression, can reduce the risk of HIV infection between 89% to 96% to uninfected sexual partners of an HIV positive individual [6–10].

The Government of Cambodia has committed to ending the AIDS epidemic by 2020 [3]. It means, by the year 2020, the annual number of new HIV infections must fall below 300 [3]. To successfully reduce the number of new HIV infections, innovative approaches are needed to reach people living with HIV who are unaware of their HIV status and link them to early diagnosis and treatment. One such approach, the Risk Tracing Snowball Approach (RTSA) has been shown to be effective in detecting new HIV cases that would otherwise not have been found [11–13]. RTSA is an adaptation of the Respondent Driven Sampling method, a form of sampling often used in bio-behavioural surveillance studies, in which study recruitment relies on peer-referrals [14]. RTSA, which is also sometimes referred to as a “Peer-Driven Intervention,” has been successfully used to increase the rate of newly undiagnosed HIV infected individuals among high-risk heterosexuals in the US [13]. It has also been found to be an effective approach to rapidly identify newly undiagnosed HIV individuals among people who use and inject drugs in Greece [12] and among heterosexual couples in China [11]. However, it has never been implemented in Cambodia context.

The main objective of this study was to evaluate the effectiveness of RTSA in increasing the rate of newly identified HIV cases among high-risk populations, including female entertainment workers (FEW), men who have sex with men (MSM), transgender (TG) women, people who inject drugs (PWID), people who use drugs (PWUD), and the general population who have social connections to key populations. The second objective was to evaluate the effectiveness of RTSA, as compared to the walk-in group, in increasing the number of HIV tests and early HIV case detection by comparing CD4 count between clients from the two groups.

## Methods

### Study settings

The RTSA was implemented between April 1 and September 30, 2016 at two clinics, Chhouk Sar I and Chhouk Sar II, in the capital city of Phnom Penh. The two clinics are operated by the Chhouk Sar Association, a non-governmental organization (NGO) established in 2010. These two clinics provide comprehensive HIV and sexual and reproductive health services, including HIV testing and counselling, ART, as well as sexually transmitted infection (STI) screening and treatment for key populations— FEW, MSM, TG women, PWID, and PWUD. These clinics also provide HIV testing services, but not ART, to the general population. Newly identified HIV positive individuals from the general population are referred to a governmental ART site.

### Intervention

The RTSA was designed by the HIV/AIDS Flagship Project [15, 16] to reach high-risk populations. We hypothesized that this intervention would increase the number of HIV tests and early HIV case detection. All clients who presented for HIV testing and counselling services at one of the two clinics were given the opportunity to serve as **“seeds”** and recruit other high-risk individuals within their personal social and sexual networks to get tested for HIV. Seeds were supplied with five coupons for use in recruiting up to five individuals from their networks. If a recruited individual (or so-called **“recruit”**) presented at either clinic with a valid coupon and screened as being “at risk” for HIV infection and completed HIV testing, the initial seed who recruited this individual would receive a financial incentive (2.5 USD). Recruits who received HIV testing and a risk assessment interview also received the same financial incentive (2.5 USD). Recruits were then offered the same opportunity as the initial seeds to recruit other high-risk peers within their social and sexual networks to get tested for HIV. This procedure was applied to all recruits, regardless of wave length, up until September 30, 2016.

### Target populations and inclusion criteria

The goal of RTSA was to reach populations at higher risk of HIV infection, but less frequently tested for HIV. This intervention included all clients presenting for HIV testing at the two selected clinics who (1) were at least 18 years old, (2) had not been tested for HIV anywhere during the previous 3 months, and (3) provided verbal informed consent which included being finger printed. They can be a FEW, MSM, TG women, PWID, PWUD, and the general population who have social connections to those key populations.

### Implementation procedures

The RTSA was built on the existing system in place at both Chhouk Sar clinics. All procedures for voluntary confidential counselling and testing (VCCT) were followed, except two more activities added to the RTSA. These two activities are described in Table 1.

**Table 1:** Process of RTSA implementation

Steps	Type of activities	
	Routine clinic processes	Additional steps for RTSA
Step 1: Clients arrived at Chhouk Sar clinic, greeted by a receptionist, and asked about services needed.	Yes	
Step 2: Clients presented for HIV testing were referred to the VCCT floor, where they received a queue number.	Yes	
Step 3a: Clients met with a counsellor for HIV pre-testing counselling.	Yes	
Step 3b: Clients who consented were registered, fingerprinted, and then interviewed using a tablet-based questionnaire.		Yes
Step 4: Clients were tested for HIV—Determine Test, Stat-Pak, and Uni-Gold [21].	Yes	
Step 5: Clients received HIV post-testing counselling. If HIV-positive, they were asked to immediately enroll into Pre-ART/ART treatment at the clinics if they self-identified as a key population. Those self-identified as the general population were referred to other Pre-ART/ART sites.	Yes	
Step 6: Clients met with coupon manager if they agreed to be part of RTSA. Coupon manager explained to seeds/recruiters on how to use the coupons and the benefits for referring.		Yes

*Abbreviation*: *RTSA* risk tracking snowball approach, *VCCT* voluntary confidential counselling and Testing

To receive the financial incentive associated with successful referrals, some seeds/recruiters presented at the clinics with the recruited individuals, while others called coupon managers to check whether their referral was a success.

It should be noted that the online fingerprint system, as mentioned in step 3b in Table 1, was an important tool to ensure that clients could not get tested for HIV more than once within a three-month period. A counsellor-administered, tablet-based questionnaire was built using the Qualtrics application. The application automatically generated the level of risk for HIV infection at the end of the interview. Clients could reject being registered into the fingerprint system or the interview. While they could still be tested for HIV, they were not part of the RTSA study.

### Questionnaire development and measures

The “risk screening tool” questionnaire was initially developed by a technical working group led by the National Centre for HIV/AIDS Dermatology and STD (NCHADS) and included key stakeholders in Cambodia. It was developed to screen for HIV risk behaviors of key populations. This study used this risk screening tool to identify individuals at risk for HIV infection.

The main outcome of interest in this study was the rate of newly identified HIV positive cases. In addition, there were three other exploratory secondary outcomes, including the absolute number of HIV tests conducted, the absolute number of newly-identified HIV positive cases, and CD4 count for those individuals tested HIV positive.

The risk assessment tool included questions used to determine key populations (FEW, MSM, TG women, PWUD, PWID) categorization, sexual behaviors with different types of sexual partners since last HIV test, or lifetime number of sexual partners if not previously been tested for HIV. Information was also collected on the history of STI symptoms, illicit drug use, hormone injection for TG women, and pregnancy and abortion for women.

### Data analyses

The rate of newly identified HIV positive cases was calculated by dividing the total number of newly identified HIV positive individuals by the total number of individuals tested for HIV during the implementation period. Chi-square test, or Fisher’s exact test when a cell count was smaller than five, were used to describe and compare characteristics between the walk-in group and the RTSA group. Factors associated with the rate of newly identified HIV positive cases were explored separately among the walk-in group and RTSA group. Bivariate and multivariable logistic regression analyses were conducted. All variables associated with HIV infection at a level of *p* < 0.25 in bivariate analyses were included in the multivariable models. STATA Version 14.1 for Windows (Stata Corp, TX, USA) was used to conduct the data analyses.

### Ethical consideration

This study was approved by the National Ethics Committee for Health Research (No.085 NECHR) of Ministry of Health in Cambodia and the FHI 360’s Protection of Human Subjects Committee (No. 590334 PHSC). All participants provided a verbal informed consent, ensuring that they understood about the voluntary and confidential participation in the study.

## Results

### Recruitment flow

As shown in Table 2, over a period of 6 months, between April 1 and September 30, 2016, a total of 721 individuals presented at the two Chouk Sar Clinics for HIV testing and counselling services. These clients were invited to serve as ‘seeds’ and recruit high-risk individuals in their social network. A total of 264 clients (36.6% of walk-in clients) agreed to serve as seeds. Approximately, 26.1% of the seeds were successful in recruiting at least one other individual within the 6 months of RTSA implementation. Of 69 networks, which seeds could refer other peers, the median number of clients per social network was six (IQR 3-9). The maximum number of clients from one network was 561 clients. During the six-month implementation, a total of 6195 coupons were distributed to seeds and recruiters; of which 1572 coupons were returned, for a coupon-return rate of 25.3%.

**Table 2:** Summary of recruitment flow

	Number	%
Period	Six months (Apr 1 2016- Sep 30 2016)	
Clients walked in for HIV testing *(walk-in group)*	721	NA
Clients invited to be seeds	721/721	100.0
Clients agreed to be seeds	264/721	36.6
Seeds referred at least one peer	69/264	26.1
Clients per network (for 69 successful seeds)		
Median (IQR)	6 (3-9)	NA
Range	2-561	NA
Coupons distributed	6195	NA
Coupons returned *(RTSA group)*	1570/6195	25.3

*Abbreviations*: *IQR* interquartile range

### Participants’ characteristics

Table 3 compares key socio-demographic characteristics of walk-in clients with those of the RTSA group. RTSA clients were significantly more likely to be male (64.5% vs. 42.4%, <0.001) and PWUD (37.7% vs. 5.6%, *p* < 0.001) compared with walk-in clients. Furthermore, RTSA clients were significantly more likely to have had a commercial sex partner since last HIV testing or lifetime if never been tested for HIV (46.4% vs. 31.5%, %, *p* < 0.001), had sex without condoms with commercial sex partners since last HIV testing or lifetime if never been tested for HIV (26.6% vs. 20.2%, *p* < 0.001), had 2 or more commercial sex partners per week since last HIV testing or lifetime if never been tested for HIV (27.1% vs. 15.3%, %, *p* < 0.001), used illicit drugs since last HIV testing or lifetime if never tested for HIV (57.7% vs. 9.5%, *p* < 0.001), and injected illicit drugs since last HIV testing or lifetime if never been tested for HIV (5.8% vs. 2.2%, *p* < 0.001).

**Table 3:** HIV testing history and risky behaviours (time frame^a^)

	Walk-in group (*N* = 609^b^)	RTSA group (*N* = 1570^b^)	
	*n* (%)	*n* (%)	*P*-value
Biological sex of participants			
Male	272 (42.4)	1014 (64.5)	<0.001
Female	370 (57.6)	558 (35.5)	
Type of population			
FEW	112 (18.4)	313 (19.9)	<0.001
MSM	29 (4.8)	138 (8.8)	
TG women	62 (10.1)	105 (6.7)	
PWUD	34 (5.6)	593 (37.7)	
PWID	7 (1.2)	76 (4.8)	
General population	365 (59.9)	345 (21.9)	
Have been tested for HIV in lifetime			
Yes	443 (72.7)	925 (58.9)	<0.001
No	166 (27.3)	645 (41.1)	
Ever had sex in lifetime			
Yes	582 (95.6)	1498 (95.4)	1.000
No	27 (4.4)	72 (4.6)	
Ever had non-commercial partner since last HIV testing or lifetime if never tested for HIV			
Yes	468 (76.8)	1285 (81.8)	<0.001
No	141 (23.2)	285 (18.2)	
Sex without a condom with non-commercial partner(s) since last HIV testing or lifetime if never tested for HIV			
Yes	442 (72.6)	1184 (75.4)	0.188
No	167 (27.4)	386 (24.6)	
Sex with commercial partner(s) since last HIV testing or lifetime if never tested for HIV			
Yes	192 (31.5)	729 (46.4)	<0.001
No	417 (68.5)	841 (53.6)	
Sex without a condom with commercial partner(s) since last HIV testing or lifetime if never tested for HIV			
Yes	123 (20.2)	417 (26.6)	0.002
No	486 (79.8)	1153 (73.4)	
Had two or more commercial partners/week since last HIV testing or lifetime if never tested for HIV			
Yes	93 (15.3)	426 (27.1)	<0.001
No	516 (84.7)	1144 (72.9)	
Ever had STI symptoms since last HIV testing or lifetime if never tested for HIV			
Yes	336 (55.2)	463 (29.5)	<0.001
No	273 (44.8)	1107 (70.5)	
Ever used drugs since last HIV testing or lifetime if never tested for HIV			
Yes	57 (9.5)	906 (57.7)	<0.001
No	543 (90.5)	664 (42.3)	
Ever injected drugs since last HIV testing or lifetime if never tested for HIV			
Yes	13 (2.2)	91 (5.8)	<0.001
No	587 (97.8)	1479 (94.2)	
Injected hormones/any substances to change your appearance (among TG women only) since last HIV testing or lifetime if never tested for HIV			
Yes	11 (18.0)	9 (8.6)	0.071
No	50 (82.0)	96 (91.4)	
Been pregnant/had a baby/ had an abortion (among women only) since last HIV testing or lifetime if never tested for HIV			
Yes	31 (31.3)	96 (31.0)	1.000
No	77 (68.7)	240 (69.0)	

*Abbreviations*: *FEW* female entertainment worker, *MSM* men who have with men, *TG women* transgender women, *PWUD* people who use drugs, *PWID* people who inject drugs

^a^Since last HIV testing if they have ever been tested for HIV or lifetime if they have never been tested for HIV

^b^The missing value (*n* = 112) in the walk-in group and (*n* = 2) in RTSA group were excluded from the analyses

RTSA clients were significantly less likely to have ever been previously tested for HIV (58.9% vs. 72.7%, *p* < 0.001) and more likely to have had STI symptoms since last HIV testing or lifetime if never been tested for HIV (29.5% vs. 55.2%, *p* < 0.001).

### Study outcomes

#### Number of HIV tests

**Fig. 1:**
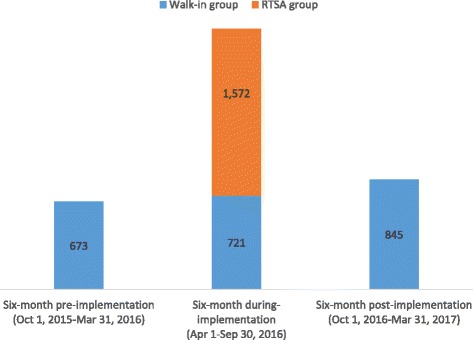
Number of individuals tested for HIV. Abbreviations: RTSA, risk tracing snowball approach

During the 6 months of RTSA implementation, a total of 721 walk-in clients and 1572 RTSA clients were tested for HIV (Fig. 1). The total number of individuals tested during the RTSA implementation (*n* = 2293) represents a 340% increase in the number of tests from the 6 months prior to the RTSA implementation (*n* = 673) and a 271% increase in the number of tests compared to the 6 months after RTSA implementation ended (*n* = 845).

#### Rate of newly identified HIV positive cases

**Fig. 2:**
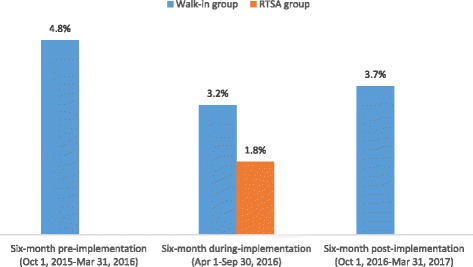
The rate of newly identified HIV positive cases. Abbreviations: RTSA, risk tracing snowball approach

As shown in Fig. 2, the rate of newly identified HIV positive cases was significantly lower among RTSA clients than that of walk-in clients during the same time frame (1.8% vs. 3.2%, *p* = 0.0445), for an odd ratio (OR) at 0.6 (95% confidence interval (CI), 0.3-0.9). Furthermore, the HIV-positive rate among RTSA was significantly lower compared to that in the 6 months prior to the RTSA implementation (1.8% vs. 4.8%, *p* < 0.001*),* for an OR at 0.4 (95% CI, 0.2-0.6) and 6 months after the RTSA implementation (1.8% vs. 3.7%, *p* = 0.006), for an OR at 0.5 (95% CI, 0.3-0.8).

#### Number of newly identified HIV positive cases

**Fig. 3:**
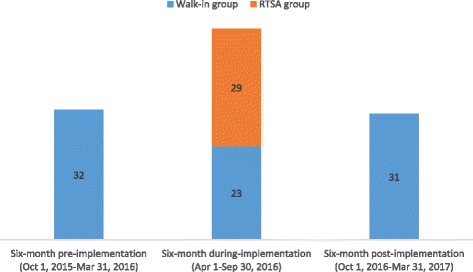
Number of newly identified HIV positive cases. Abbreviations: RTSA, risk tracing snowball approach

Despite the lower rate of newly identified HIV positive cases among RTSA group, the highest number of newly identified HIV positive cases was found during the RTSA implementation period compared to that in the 6 months prior to and 6 months after the RTSA implementation. While only 32 positive cases were found within the 6 months prior to the RTSA implementation and 31 cases in the 6 months after the RTSA implementation, a total of 52 cases were found during the 6 months of the RTSA implementation (Fig. 3).

#### CD4 count

CD4 test results were available for only 28 out of 52 newly HIV diagnosed individuals (11 from the RTSA group and 17 from the walk-in group). Although not statistically significant, the mean CD4 count of newly identified HIV positive cases in the RTSA group was almost 200 cells/mm^3^ higher than that among those in the walk-in group (540 ± 271 vs. 355 ± 350).

#### Recruiters of newly identified HIV positives

As shown in Table 4, HIV-negative clients recruited a total of 1532 clients (out of 1572 clients in RTSA). The rate of newly identified HIV positive cases among those who were recruited by HIV negative clients was 1.8%. Clients who were already aware of their HIV-positive status recruited 19 clients and generated 5.2% rate of newly identified HIV positive cases. However, clients who were newly identified as HIV positive recruited 21 clients, but never recruited other newly identified HIV positive clients.

**Table 4:** Recruiters of newly identified HIV positive cases

Recruiters	Recruits			
	Negative	Positive-newly identified	Positive-already aware status^a^	Total
Negative	97.5%	1.8%	0.7%	100% (1532)
Positive-newly identified	95.7%	0.0%	4.5%	100% (21)
Positive-already aware status	94.7%	5.2%	0.0%	100% (19)

^a^All of HIV positive clients who were already aware their HIV positive status admitted during post-test counselling. The main reason for re-testing was because they wanted to be re-enrolled in pre-ART/ART services after having lost to follow up at other sites

**Fig. 4:**
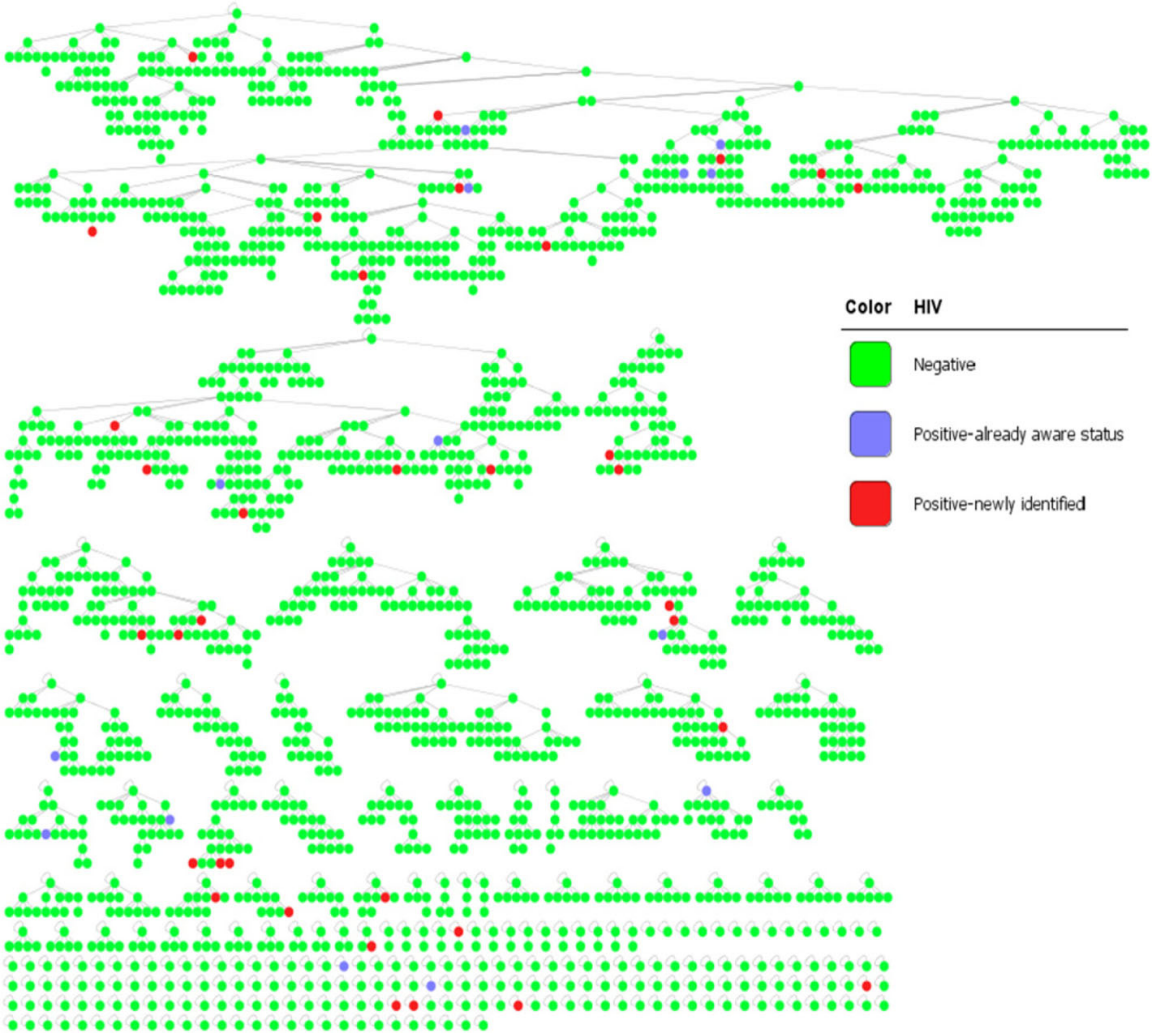
Recruitment graph

Translated from Table 4 above, we visualized the network in a graph as shown in Fig. 4 for a better understanding of how the networks were formed. Red nodes represent newly identified HIV clients; green nodes represent HIV-negative clients; and blue nodes represent clients who were already aware of their HIV-positive status. In most cases, recruitment stops when a client is newly identified as HIV positive.

#### Factors associated with HIV infection

Table 5 shows the rate of newly identified HIV positive cases and its unadjusted associated factors for the walk-in group and RTSA group, separately.

**Table 5:** Bivariate results of factors associated with HIV infection

	Walk-in group (*N* = 609)		RTSA group (*N* = 1570)	
	HIV rate (*N* = 14), *n* (%)	OR (95% CI)	HIV rate (*N* = 26), n (%)	OR (95% CI)
Type of population				
FEW	4 (3.6)	**4.5 (1.0;20.2)**	10 (3.2)	3.8 (1.0;13.9)*
MSM	2 (6.9)	8.7 (1.4;54.6)*	2 (1.5)	1.7 (0.3;10.1)
TG women	5 (8.1)	10.4 (2.4;44.5)*	2 (1.9)	2.2 (0.4;13.4)
PWUD	0 (0.0)	NA	9 (1.5)	1.7 (0.;6.5)
PWID	0 (0.0)	NA	0 (0.0)	NA
General population	3 (0.8)	Ref.	3 (0.9)	Ref.
Previously tested for HIV				
Yes	8 (1.8)	0.5 (0.2;1.4)	17 (1.8)	1.3 (0.6;30)
No	6 (3.6)	Ref.	9 (1.4)	Ref.
Ever had non-commercial partner since last HIV testing or lifetime if never tested for HIV				
Yes	**9 (1.9)**	0.5 (0.2;1.6)	21 (1.6)	0.9 (0.4;2.5)
No	**5 (3.6)**	**Ref.**	5 (1.8)	Ref.
Sex without a condom with non-commercial partner(s) since last HIV testing or lifetime if never tested for HIV				
Yes	7 (1.6)	0.4 (0.1;1.0)	6 (1.6)	1.1 (0.5;2.7)
No	7 (4.3)	Ref.	20 (1.8)	Ref.
Sex with commercial partner(s) since last HIV testing or lifetime if never tested for HIV				
Yes	7 (3.7)	2.2 (0.8;6.3)	15 (2.1)	1.6 (0.7;3.5)
No	7 (1.7)	Ref.	11 (1.3)	Ref.
Sex without a condom with commercial partner(s) since last HIV testing or lifetime if never tested for HIV				
Yes	5 (4.1)	2.2 (0.7;6.8)	10 (2.4)	1.7(0.8;3.9)
No	9 (1.9)	Ref.	16 (1.4)	Ref.
Had two or more commercial partners per week since last HIV testing or lifetime if never tested for HIV				
Yes	3 (3.2)	1.5 (0.4;5.6)	7 (1.7)	1.0 (0.4;2.4)
No	11 (2.1)	Ref.	19 (1.6)	Ref.
Experienced STI symptoms since last HIV testing or lifetime if never tested for HIV				
Yes	7 (2.1)	0.8 (0.3;2.3)	12 (2.6)	2.1 (0.9;4.5)
No	7 (2.6)	Ref.	14 (1.3)	Ref.
Ever used drugs since last HIV testing or lifetime if never tested for HIV				
Yes	2 (3.6)	1.7 (0.4;8.1)	15 (1.7)	1.0 (0.5;2.0)
No	11 (2.1)	Ref.	11 (1.7)	Ref.
Ever injected drugs since last HIV testing or lifetime if never tested for HIV				
Yes	1 (7.7)	4.0 (0.5;33.0)	0 (0.0)	NA
No	12 (2.1)	Ref.	26 (1.8)	NA
Injected hormones/any substances to change your appearance (among TG women only) since last HIV testing or lifetime if never tested for HIV				
Yes	1 (9.1)	1.2 (0.1;11.4)	0 (0.0)	NA
No	4 (8.0)	Ref.	2 (2.1)	NA
Been pregnant/had a baby/ had an abortion (among women only) since last HIV testing or lifetime if never tested for HIV				
Yes	0 (0.0)	NA	2 (2.1)	0.6 (0.1;2.6)
No	4 (5.2)	NA	8 (3.7)	Ref.

*Abbreviations*: *OR* odds ratio, *CI* confidence interval, *Ref* reference group, *FEW* female entertainment worker, *MSM* men who have with men, *TG women* transgender women, *PWUD* people who use drugs, *PWID* people who inject drugs; (*) *P*-value < 0.05

In the walk-in group, using the general population as the reference group, the rate of newly identified HIV positive cases was significantly higher among MSM (unadjusted OR 8.7, 95% CI 1.4, 54.5), and TG women (unadjusted OR 10.4, 95% CI 2.4, 44.5).

In the RTSA group, using the general population as the reference group, the rate of newly identified HIV positive cases was significantly higher among FEW (unadjusted OR 3.8, 95% CI 1.0, 13.9). Unlike the walk-in group, the rate of newly identified HIV positive cases was not statistically different for MSM and TG women when compared to the general population, although the estimated effects were in the same direction.

Table 6 presents the results from multivariable logistic regression analyses. After adjustment for other variables in the models, in the walk-in group, a significantly higher rate of newly identified HIV positive cases was found among TG women (adjusted OR 15.2, 95% CI 3.1, 75.2), MSM (adjusted OR 10.4, 95% CI 1.4, 77.0), and a significantly lower rate was found among clients who reported previouly tested for HIV (adjusted OR 0.3, 95% CI 0.1, 0.9) compared to their comparison group. Unlike the walk-in group, in the RTSA group, a significantly higher rate of newly identified HIV positive cases was found among FEW compared to that in the general population (adjusted OR 2.7, 95% CI 1.1, 7.1).

**Table 6:** Adjusted factors associated with HIV infection

	Walk-in group	RTSA group
	(N = 609)	(N = 1570)
	Adjusted OR	Adjusted OR
	(95% CI)	(95% CI)
Type of population		
FEW	5.2 (0.6;45.2)	5.2 (1.0;28.9)
MSM	10.4 (1.4;77.0)*	2.1 (0.3;14.4)
TG women	15.2 (3.1;75.2)**	2.7 (0.4;17.9)
PWUD	NA	2.3 (0.6;9.2)
PWID	NA	NA
General population	Ref.	Ref.
Previously tested for HIV		
Yes	0.3 (0.1;0.9)*	1.2 (0.5;2.7)
No	Ref.	Ref.

*Abbreviations*: *OR* odds ratio, *CI* confidence interval, *Ref* reference group, (*) *P*-value < 0.05, (**) *P*-value < 0.001

## Discussion

This study found that the rate of newly identified HIV positive cases generated by RTSA was significantly lower compared to the rate in the traditional walk-in approach. However, we found that the absolute number of HIV cases detected during RTSA implementation (RTSA and walk-in approach combined) was much higher than that in the six-month period prior to the RTSA implementation and the six-month period after the RTSA implementation ended. This finding indicates that a combination of RTSA and a traditional walk-in approach may increase new case detection among high-risk populations, especially key populations.

The lower rate of newly identified HIV positive cases among the RTSA clients than that in the walk-in clients is not surprising. Individuals who walked in to get tested are more likely to do so because of their self-perception of their risky behaviors, or because they felt sick. It is also plausible that when more individuals are tested, the HIV rate will naturally go down.

Even though the rate of newly identified HIV positive cases for the RTSA group was lower than the rate obtained from the clinics’ routine data (walk-in group), this rate should still be considered acceptable for four reasons. First, the combination of the routine walk-in testing and RTSA during RTSA implementation boosted the absolute number of HIV new case detection from 32 in the six-month period prior to the RTSA implementation to 52 cases in the six-month period of RTSA implementation. Second, CD4 count in RTSA group was higher than that in the walk-in group. This may infer that RTSA leads to earlier detection of people living with HIV. Third, the rate of newly identified HIV positive cases in RTSA group was much higher than the rate of newly identified HIV positive cases reported nationwide in programs using an outreach HIV testing approach among key populations—FEW, MSM, TG women, and PWID-- which has never reached more than 1.0% since the program implementation was started in 2013 [15, 16]. Fourth, the rate of newly identified HIV positive cases in RTSA group is comparable to the national HIV prevalence in each key population: FEW (3.8% vs. 4.6%), MSM (1.7% vs. 2.3%), TG women (2.2% vs. 5.9%), and PWUD (1.7% vs. 4.1%), except PWID (0% vs. 25%) [17–20].

Other than being in a high-risk group (TG women, MSM) and experiencing tested for HIV, our study did not find any factors associated with the rate of newly identified HIV positive cases in either groups—walk-in and RTSA. The non-significant differences may be explained by the following reasons. First, as with any other self-reporting measures, risky behaviors may have been underreported due to social desirability. This was also observed among the walk-in group by counsellors. Thus, levels of risks may have been misclassified. In contrast, the RTSA group may have over-reported their risks to make their recruiters eligible for financial incentives. It should be reminded that all recruited individuals can get HIV testing and receive 2.5 USD transportation compensation regardless their risk status and HIV testing result. However, seeds or recruiters can only get the incentive when their recruits were screened at risk for HIV infection. Second, our questionnaire has never been validated. We may not have asked the right questions to identify risks. And third, the small sample size may have contributed to the non-statistically significant associations. Regardless what might be the reasons, using a risk-screening tool to identify risky clients may have low validity, especially when it confounded by the use of monetary incentives. Excluding those who reported low risk to HIV infection may also have influenced our ability to further elucidate risk factors.

Theoretically, people are more likely to refer those who are similar to them [14]. This theory is not necessarily true considering HIV testing results in this study. HIV negative clients may be happier after getting the test result and might be willing to refer their peers. In contrast, positive clients were more likely to stop the recruitment after they learned about their HIV-positive status. As shown in the network graph, most of the positive clients did not recruit anyone else to join the study. This nature has blocked the power of network referral. If HIV-positive clients could recruit more people, they may recruit their sexual partners and their peers who had similar risks. However, newly detected HIV-positive individuals need some time to stabilize their emotional condition first, or they will never recruit other people because they do not want others to suspect or learn about their HIV status.

The strength of this study is the use of well-managed patients tracking system in Chhouk Sar clinics where the study was conducted. The online fingerprint system can track HIV test interval of individual clients. Clients could not be tested multiple times at one or another Chhouk Sar clinic within 3 months. However, our study also has several limitations. First, we could only trace clients in our system. We could not manage if they were tested elsewhere. Second, the self-reported risky behaviors may not be reliable due to the use of monetary incentives. However, identifying sexual risky behaviors was not the main objective of the study. Rather, it was the rate of newly identified HIV positive cases that detected by three-test algorithm —Determine Test, Stat-Pak, and Uni-Gold [21]. Third, the identification of newly detected cases was based on counsellors’ skills. In this sense, we cannot deny the possibility of inclusion of formerly detected HIV clients in the newly identified HIV positive group. However, because all newly identified HIV-positive clients were closely followed-up until they got enrolled in the treatment, only minor discrepancies were likely to happen. Lastly, our study ended up with inconclusive result whether the RTSA is cost-effective because the study design did not allow further investigation on this important aspect. To address this shortcoming, cost-effectiveness modelling should be conducted. This kind of study will be able to assess the number of people who will be prevented from HIV infection resulting from early detection by RTSA, and unit cost to invest to keep a person uninfected with HIV per year – known as Disability-Adjusted Life Year (DALY) [22].

## Conclusions

The rate of newly identified HIV positive cases among the RTSA group was lower than that found within the walk-in group, but the inclusion of RTSA method in addition to the traditional walk-in method boosted new HIV case detection. The higher CD4 counts found in the RTSA group compared to that in the walk-in group may reveal that RTSA could detect HIV cases earlier than walk-in approach could do. Further research is needed to understand whether RTSA is a cost-effective intervention to prevent ongoing spread of the HIV.

## Abbreviations

AIDS: Acquired immune deficiency syndrome
ART: Antiretroviral therapy
CD4: Cluster of differentiation 4
CI: Confidence interval
FEW: Female entertainment workers
HIV: Human immunodeficiency virus
IQR: Interquartile range
MSM: Men who have sex with men
NECHR: National Ethics Committee for Health Research
NGO: Non-governmental organization
PHSC: Protection of Human Subjects Committee
PWID: People who inject drugs
PWUD: People who use drugs
RTSA: Risk tracking snowball approach
STI: Sexually transmitted infection
TG: Transgender women
US: United State
VCCT: Voluntary confidential counselling and testing
